# Adult Human Brain Neural Progenitor Cells (NPCs) and Fibroblast-Like Cells Have Similar Properties In Vitro but Only NPCs Differentiate into Neurons

**DOI:** 10.1371/journal.pone.0037742

**Published:** 2012-06-04

**Authors:** Thomas In-Hyeup Park, Hector Monzo, Edward W. Mee, Peter S. Bergin, Hoon H. Teoh, Johanna M. Montgomery, Richard L. M. Faull, Maurice A. Curtis, Mike Dragunow

**Affiliations:** 1 Department of Pharmacology and Clinical Pharmacology, Faculty of Medical and Health Sciences, The University of Auckland, Auckland, New Zealand; 2 The Centre for Brain Research, Faculty of Medical and Health Sciences, The University of Auckland, Auckland, New Zealand; 3 Department of Anatomy and Radiology, Faculty of Medical and Health Sciences, The University of Auckland, Auckland, New Zealand; 4 Department of Neurosurgery, Auckland City Hospital, Auckland, New Zealand; 5 Department of Neurology, Auckland City Hospital, Auckland, New Zealand; 6 Labtests, Auckland, New Zealand; 7 Department of Physiology, Faculty of Medical and Health Sciences, The University of Auckland, Auckland, New Zealand; University of North Dakota, United States of America

## Abstract

The ability to culture neural progenitor cells from the adult human brain has provided an exciting opportunity to develop and test potential therapies on adult human brain cells. To achieve a reliable and reproducible adult human neural progenitor cell (AhNPC) culture system for this purpose, this study fully characterized the cellular composition of the AhNPC cultures, as well as the possible changes to this *in vitro* system over prolonged culture periods. We isolated cells from the neurogenic subventricular zone/hippocampus (SVZ/HP) of the adult human brain and found a heterogeneous culture population comprised of several types of post-mitotic brain cells (neurons, astrocytes, and microglia), and more importantly, two distinct mitotic cell populations; the AhNPCs, and the fibroblast-like cells (FbCs). These two populations can easily be mistaken for a single population of AhNPCs, as they both proliferate under AhNPC culture conditions, form spheres and express neural progenitor cell and early neuronal markers, all of which are characteristics of AhNPCs *in vitro.* However, despite these similarities under proliferating conditions, under neuronal differentiation conditions, only the AhNPCs differentiated into functional neurons and glia. Furthermore, AhNPCs showed limited proliferative capacity that resulted in their depletion from culture by 5–6 passages, while the FbCs, which appear to be from a neurovascular origin, displayed a greater proliferative capacity and dominated the long-term cultures. This gradual change in cellular composition resulted in a progressive decline in neurogenic potential without the apparent loss of self-renewal in our cultures. These results demonstrate that while AhNPCs and FbCs behave similarly under proliferative conditions, they are two different cell populations. This information is vital for the interpretation and reproducibility of AhNPC experiments and suggests an ideal time frame for conducting AhNPC-based experiments.

## Introduction

The existence of adult neural stem cells in the rodent brain was reported in the 1960s [1], [2] and by the early 1990s, they were successfully isolated, propagated and differentiated into neurons *in* *vitro* [3]. Subsequent studies in the human brain also found neural stem/progenitor cells [4], herein referred to as adult human neural progenitor cells (AhNPCs), and like our mammalian counterparts, they were largely restricted to two neurogenic regions; the subventricular zone (SVZ) lining the lateral ventricle and the subgranular zone (SGZ) of the hippocampus [3], [4]. Under physiological conditions, AhNPCs of the SVZ and SGZ proliferate and migrate to the olfactory bulb and the dentate gyrus respectively to give rise to neurons and astrocytes [5], [6], [7]. A number of reports also showed that AhNPCs were capable of being cultured *in vitro* from both autopsy and biopsy human brain tissue [8], [9], [10], [11], [12], [13], [14], [15], [16]. Most of these studies utilized the neurosphere (NS) culture method [3], [17], and provided evidence for self-renewal through 5-bromo-2-deoxyuridine (BrdU) incorporation studies [8] and single AhNPC clonal expansion studies [10], [16]. Furthermore, NS-derived cells differentiated into antigenically [11], [13] and physiologically [12], [16] identifiable neurons and glia, fulfilling the criteria for neural progenitor cells (NPCs) *in* *vitro*. More recently, techniques have been established for culturing AhNPCs as a monolayer, removing the laborious process of NS formation and allowing characterization of individual AhNPCs in culture dishes [9], [11], [15]. These pioneering studies have addressed many important aspects of AhNPC cultures and have laid the foundation for future experiments. However, AhNPC culture studies are still faced with issues associated with reliable characterization and hence, experimental reproducibility. For example, most studies have found AhNPCs to only be present in the neurogenic regions [8], [11], [18], while some studies report their presence in classically non-neurogenic regions [14], [19], [20]. There are also discrepancies in the literature regarding the mitotic capacity of AhNPCs *in vitro*, as some report limited (<6 months) self-renewal [13], [16], [20], whilst others have reported successful long-term (>12 months) cultures [9], [11], [15]. Further complicating the interpretation of these previous reports is the large degree of heterogeneity observed with primary human brain cell cultures, including a fibroblast-like cell (FbC) population that also divides *in* *vitro* [21]. It is possible that FbCs have contaminated cultures presented in previous publications, thus accounting for some of the discrepancies in the literature. It is also possible that these FbCs are, in fact, another source of NPCs in the brain [22].

For these reasons, we studied the proliferating cell types grown from adult human brain tissue to compare their properties and determine whether FbCs and AhNPCs are two distinct populations or arise from a common source. Here, we demonstrate that the neurogenic regions of the adult human brain can give rise to two different populations of mitotic cells that have remarkable similarities during their proliferative states *in vitro*. However, AhNPCs were limited to the neurogenic regions and gave rise to physiologically active neurons, while the FbCs could also be isolated from the non-neurogenic cortical regions but failed to differentiate into mature neuronal cell types. Most importantly, we demonstrate the relatively limited proliferative ability of the AhNPCs compared to the FbCs and suggest a time frame in which AhNPC experiments should be conducted for accurate interpretation of *in vitro* experiments of adult human neurogenesis.

## Results

The initial isolation of cells from the adult human hippocampus (HP) and the surrounding SVZ resulted in a heterogeneous population of cells in culture. They comprised of post-mitotic astrocytes, neurons and microglia, and the mitotic AhNPCs and FbCs. Post-mitotic astrocytes were positive for glial fibrillary acidic protein (GFAP) and negative for the cell cycle marker ki67 (Figure S1 A-C). Neuronal cells were βIII-tubulin positive and ki67 negative (Figure S1 D-F) and microglial cells were CD45 and PU.1 positive (Figure S1 G-I).

**Figure 1:**
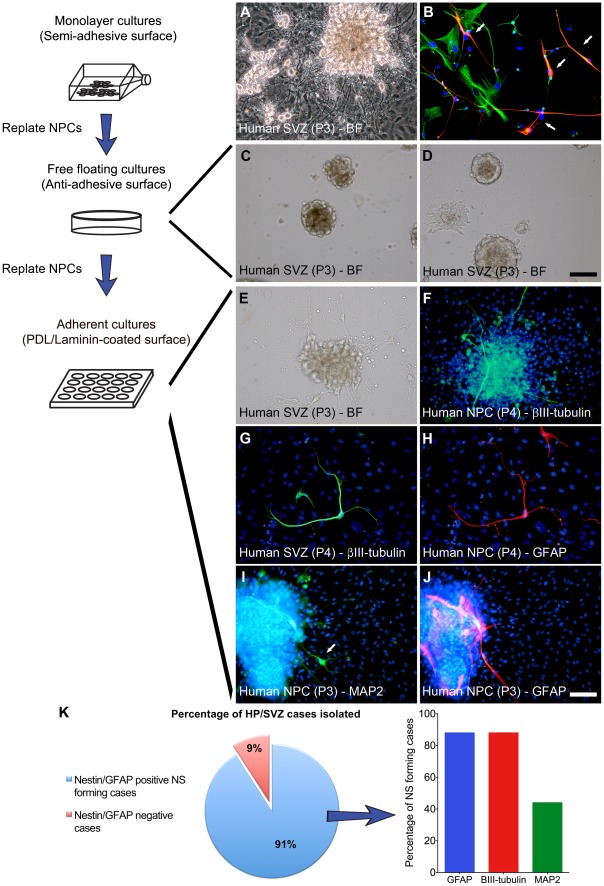
A population of mitotically active cells from biopsy specimens show NPC-like characteristics. (**A**) Passage 3 of isolated cells grown as monolayer cultures in serum-free NPC proliferation media. The culture is composed of phase-bright dividing bipolar shaped cells and spontaneously forming and differentiating NSs. **(B)** Arrows indicate cells co-expressing Nestin (green) and GFAP (red). (**C-D**) Cells passaged and plated onto non-adhesive petri-dishes formed numerous NSs. (**E**) Represents a bright-field image of a differentiating NS and (**F**) shows these cells expressing βIII-tubulin (green). (**G-H**) βIII-tubulin and GFAP co-localized ‘asteron’ cells and (**I-J**) show single labelled neuronal MAP2 (arrow) and astroglial GFAP positive cells. (**K**) Pie graph showing the percentage of our human brain culture cases that generated AhNPCs (i.e. produced neurospheres, co-expressed Nestin and GFAP). The bar graph indicates the percentage of the AhNPC-positive cases that gave rise to βIII-tubulin, GFAP and MAP2 positive cells when differentiated. Scale: 100 µm.

The mitotic population could be divided into two groups based on their morphology and antigenicity. The first mitotic population consisted of GFAP and Nestin positive NPCs that also expressed ki67 (Figure S2 A-F). These NPCs also incorporated BrdU and co-expressed PSA-NCAM and βIII-tubulin (Figure S2 G-I). The second mitotic population was also Nestin and ki67 positive, but did not express GFAP (Figure S2 J-L). Instead, they were immunoreactive for the collagen synthesizing enzyme prolyl-4-hydroxylase (P4H) and the extracellular matrix protein fibronectin (Figure S2 M-P). Morphologically, they were generally flat and had a wide-spread cytoplasm, which together, led us to classify them as FbCs. Every region tested, including the SVZ, gave rise to neurons, microglia, astrocytes, and FbCs. However, AhNPCs could only reliably be cultured from the SVZ regions, with 10 out of the 11 cases resulting in identifiable NPCs (Figure 1 K). AhNPCs were Nestin and GFAP positive (Figure 1 B), proliferated as a monolayer on un-coated tissue culture flasks (semi-adhesive) and gave rise to spontaneous neurospheres (NSs; Figure 1 A). When differentiated, over 80% of these cases resulted in βIII-tubulin and GFAP positive cells (Figure 1 K), indicating that the majority of NS forming cases show neurogenic potential. When NSs were dissociated into single cells and plated onto non-adhesive surfaces, they were capable of forming secondary and tertiary NSs (Figure 1 C-D), although with diminishing efficiency. To induce neuronal differentiation, spheres were plated onto adhesive surfaces (PDL/Laminin) and medium was changed to a no or low serum containing media devoid of mitotic growth factors. Adherent NSs rapidly extended processes and cell migration soon followed (Figure 1 E), and 35% of the cells expressed the immature neuronal marker βIII-tubulin (Figure 1 F, 3 E). The same population was also immunoreactive for the mature neuronal marker MAP2 and the astroglial marker GFAP (Figure 1 I–J), indicating that AhNPCs can give rise to at least two cell lineages of the CNS *in vitro.* Furthermore, intermediate cell types co-expressing βIII-tubulin and GFAP were observed (Figure 1 G–H).

**Figure 2:**
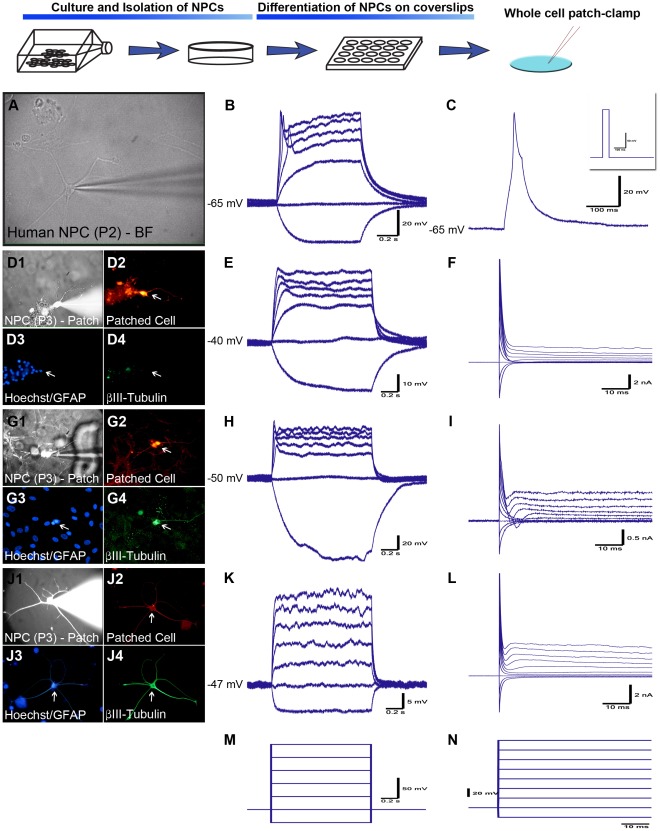
NPCs differentiated on PDL/Laminin-coated coverslips give rise to cells showing neurophysiological properties. (**A-C**) Bright field image of a differentiated NPC during patch-clamp recordings and the electrophysiological responses to multiple and single current steps respectively (left to right). This cell had a RMP of −65 mV and fired an immature action potential (AP) in response to depolarizing current. The insert in (C) was the stimulus protocol. (**D–F**) AhNPC differentiated for 5 days. This cell did not express βIII-tubulin or GFAP, but had clear neurite processes (D1–4). It exhibited a small depolarization and a small inward current in response to depolarizing stimuli (**E–F**). (**G–L**) AhNPCs recorded after 3–4 weeks of differentiation. (**G–I**) Recording from a βIII-tubulin positive, GFAP-negative cell (D3–D4) that fired an immature APs (H), and exhibited small, fast-inactivating inward and slow-inactivating outward currents (I). (J1–4) An ‘asteron’ cell expressing both GFAP and βIII-tubulin. These cells failed to elicit any active voltage or current responses to depolarizing stimuli (**K-L**). (**M-N**) The stimulus protocols for current clamp (M) and voltage clamp (N). All traces are representative recordings from 5 different biopsy cases that were conducted from either passage 2 or 3 cells.

The differentiated AhNPCs with neurite-bearing morphology from 5 separate cases were subjected to electrophysiological studies to confirm their functional differentiation. During the first week of differentiation, neuron-like cells exhibited a depolarized resting membrane potential (RMP) and a high membrane resistance (R_m_) of −45 mV±3.2 mV and 654±210 MΩ (n = 7), respectively. When tested for active membrane properties, 2 of the 7 cells responded with a small depolarization (Figure 2 D–F). Continued differentiation (3–4 weeks) resulted in RMP and R_m_ values sitting at near physiological levels of −65±2.1 mV and 195±50 MΩ (n = 10), respectively. Active membrane properties also matured with 3 out of the 10 cells (30%) firing an immature action potential (iAP) in response to the depolarizing stimuli (represented by Figure 2 A–C & G-I). These APs were accompanied by a relatively fast inactivating inward and a slow inactivating outward current. The same AP firing cells exhibited βIII-tubulin staining (Figure 2 G1–4). The majority of the fiber-bearing cells elicited little or no active membrane responses to depolarizing currents or voltages (Figure 2 J–L) and generally had a hyperpolarized RMP and a lower R_m_. When separately grouped into iAP firing and non-firing cells, the iAP firing cells had an average RMP and R_m_ of −61.7±3.0 mV and 333±100 MΩ, respectively, while the non-firing cells showed glial-like properties with a RMP of −67.5±1.6 mV and R_m_ of 118±13 MΩ (P<0.05). The cells that expressed both GFAP and βIII-tubulin (Figure 2 J–L) did not fire an iAP but exhibited a slight inward current under large depolarizing stimuli. These results demonstrated that our AhNPC cultures gave rise to at least 2 brain cell populations *in vitro,* neurons and astrocytes.

**Figure 3:**
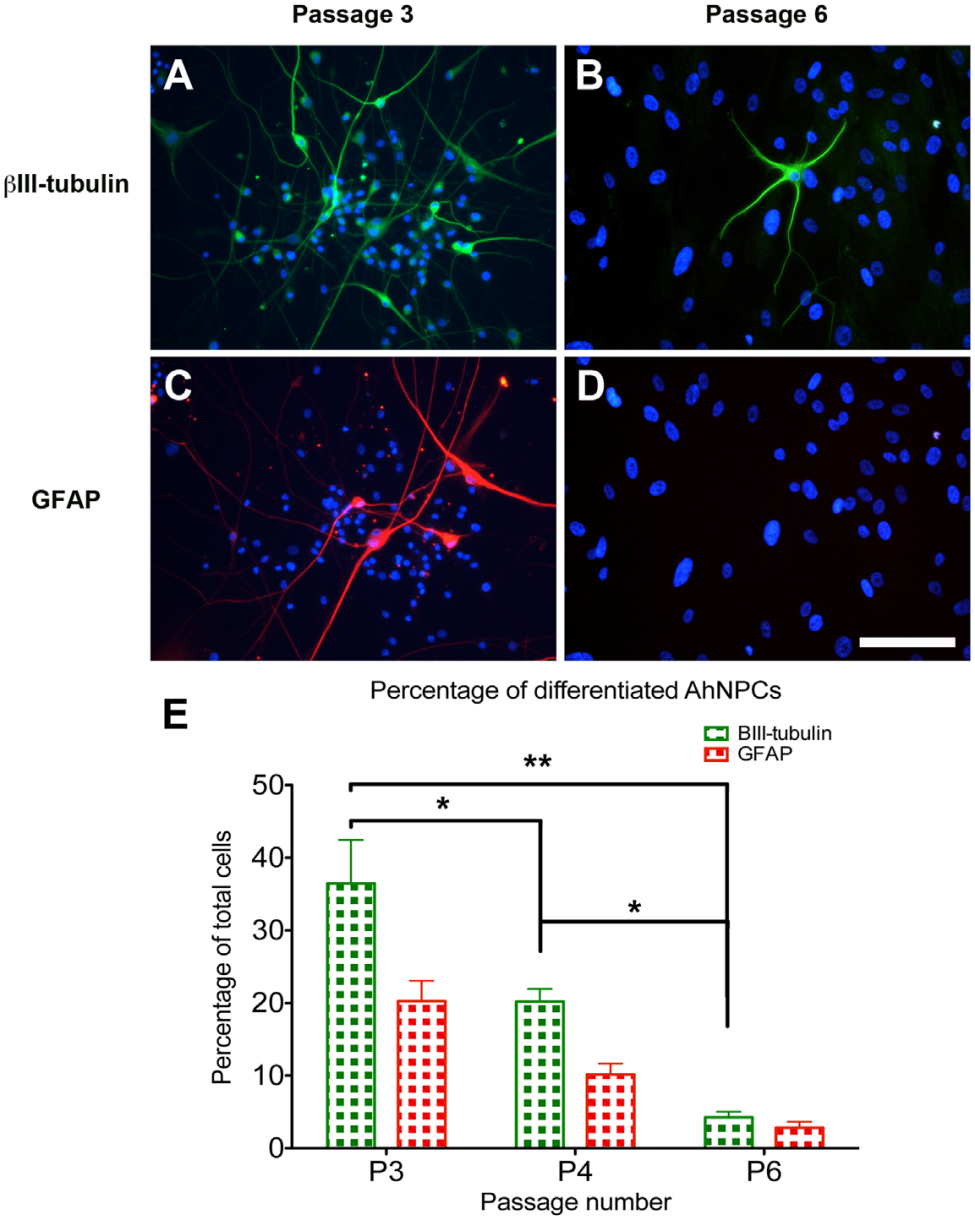
Photomicrograph and bar graph illustrating the decrease in neurogenic capabilities observed with increased culture duration. (**A**, **C**) Illustrates the extent of neuronal (βIII-tubulin) and astrocytic (GFAP) differentiation seen after 3 weeks of differentiating AhNPCs from cultures at passage 3. (**B**, **D**) Illustrates the same parameters when AhNPC cultures from passage 6 were differentiated. These images demonstrate the dramatic decline in the neurogenic capabilities of the AhNPC cultures with increased culture duration. (**E**) Quantification of the percentage of differentiated neurons and astrocytes derived from the 3 independent cases using automated images analysis. Statistical analysis using one-way Anova demonstrates the significant reduction in the percentage of βIII-tubulin cells (*  =  P<0.05, **  =  P<0.01).

Greater than 60% of our cultures continued to proliferate for longer than 8–10 months (>6 passages), formed NSs and expressed the NPC marker Nestin (Figure 1 B). However, the ability of these cultures to generate neurons and astrocytes (their neurogenic ability) deteriorated with successive passages, reaching insignificant levels by 5–6 months (∼ 6 passages; Figure 3). The morphological composition of the cultures also progressively changed, going from phase bright monolayer and sphere-dominated cultures (<5 months) to relatively homogenous bipolar monolayer cultures with limited sphere formations (>6 months). These long-term cultured cells showed strong resemblance to the FbCs reported previously by our laboratory [21], except for their bipolar morphology and the level of Nestin expression.

**Figure 4:**
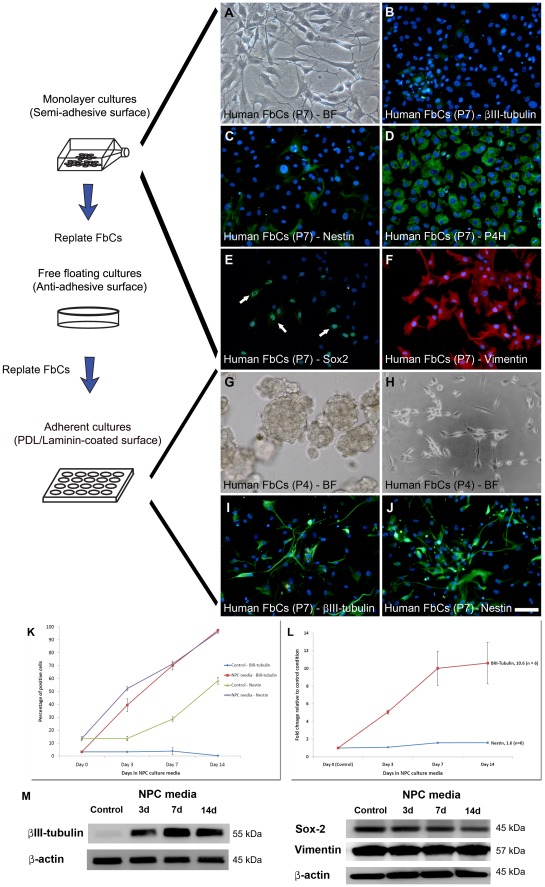
The FbCs isolated from biopsy specimens show NPC-like properties under NPC culture conditions but failed to differentiate into functional neurons. (**A**) Bright-field (BF) image showing the distinct morphology of the FbCs. They exhibited a flat and diffuse cytoplasm and were generally phase-dark. These cells showed characteristics of fibroblasts, as they lack the expression of neuronal βIII-tubulin (**B**), but had high levels of collagen synthesizing enzyme prolyl-4-hydroxylase (P4H; **D**) and Vimentin (**F**). However, they also expressed stem cell-like markers such as Nestin (**C**) and Sox-2 (**E**). Furthermore, when cultured under neurosphere-forming conditions, FbCs also formed spheres (**G**) that could be passaged for at least 2 – 3 passages. When FbCs were cultured in NPC culture conditions, their morphology changed to phase-bright polar shaped cells (**H**) and started expressing high levels of βIII-tubulin (**I**) and up-regulated their expression of Nestin (**J**). In agreement with immunostaining results, quantitative image analysis revealed NPC conditions gradually increased the percentage of FbCs expressing Nestin and βIII-tubulin at a detectable level (**K**). When compared to the control astrocytic culture condition, these differences were significant (P<0.01). Q-RT-PCR data also showed βIII-tubulin mRNA levels increased by over 10 fold by 14 days of exposure to NPC conditions (**L**; P<0.01). Due to the relatively high basal levels of Nestin in the control cells, the relative increase in Nestin was only 1.5 fold, but this still reached significance (**L**; P<0.01). Finally, western blots validated many of the above observations, as in control conditions, they showed βIII-tubulin at near un-detectable levels, but increased significantly during the first 3 days in NPC media and continued to increase till day 7 (**M**). Sox-2 and Vimentin were highly expressed in control and NPC media-induced FbCs (**M**). These results were consistently observed in all the cases tested (n = 5). Scale: 100 µm.

To elucidate whether the differences in morphology and the stronger expression of Nestin was due to the NPC culture conditions, FbCs cultured in serum-containing culture conditions from the temporal cortex [21] were cultured in our serum-free NPC proliferation media. NPC culture conditions resulted in the FbCs showing markedly different characteristics to those cultured in serum containing conditions. Immunostaining revealed that the NPC marker Nestin, although expressed at detectable levels in FbCs cultured in serum containing media, further increased during the 14 days of NPC media induction (Figure 4 C & J). This increase was quantified using image analysis and q-RT-PCR and showed a 1.5 to 2 fold change in expression (P<0.01; Figure 4 L). Furthermore, βIII-tubulin, which was not present at immunologically detectable levels in FbCs cultured in serum containing media (Figure 4 B), increased significantly during the 2 weeks in NPC media (Figure 4 I) with image analysis and q-RT-PCR data indicating a larger than 10 fold increase in expression (P<0.01; Figure 4 K–L). Western blot results were consistent with the above (Figure 4 M). NPC media-exposed FbCs also changed their morphology to resemble cells those from the AhNPC cultures with bipolar processes and phase-bright somas (Figure 4 H). Furthermore, when the FbCs were cultured in NS forming conditions (i.e. plated onto non-adhesive surfaces at clonal densities in NPC media), a large number of cells formed primary spheres (Figure 4 G) that were capable of forming secondary spheres from their dissociated cells. These results were confirmed in all of 5 trialed independent cases and demonstrated the close phenotypic resemblance between the FbCs and the cells present in our AhNPC cultures. However, like the cells found during the later passages (>6) of our AhNPC cultures, the FbC-derived spheres were not capable of undergoing full neuronal or astrocytic differentiation, as they gave rise to cells expressing βIII-tubulin but not MAP2 or GFAP. They also failed to give rise to cells exhibiting neurophysiological properties with average RMP of the cells sitting at –6.5±6 mV and R_m_ 500±300 MΩ (n = 5), and failed to elicit any form of active membrane properties (data not shown). Table 1 summarizes these findings, which indicate that the majority of the mitotic cells present in the later passages of a primary AhNPC cultures are likely to be the FbCs and not NPCs.

**Table 1:** Summary of the characteristics of cells isolated from adult human SVZ/hippocampus.

Cell types	Proliferation	Sphere formation	Positive Markers	Differentiation
**Neurons**	No	No	βIII-tubulin, MAP-2ab	N/A
**Astrocyte**	No	No	GFAP	N/A
**Microglia**	Limited ki67	No	CD45, PU1, HLA-DR	N/A
**NPCs**	Yes (BrdU, ki67)	Yes	Nestin, Sox-2, GFAP, Vimentin,PSA-NCAM, βIII-tubulin	βIII-tubulin, MAP-2ab, GFAP, Neurophysiology
**FbCs**	Yes (BrdU, ki67	Yes	Nestin, Sox-2, Vimentin, βIII-tubulin,Prolyl-4-hydroxylase, Fibronectin, S100A4,α-SMA, PDGFRβ	βIII-tubulin, Fibroblast, Fibronectin, No neurophysiology

N/A  =  Not available due to negative results.

**Figure 5:**
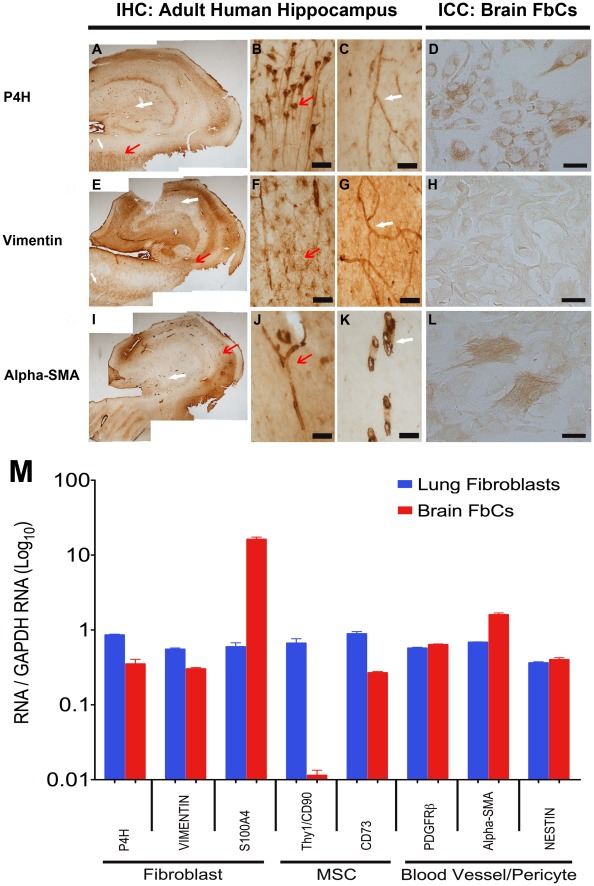
The brain-derived FbCs isolated from biopsy specimens are likely to be of neurovasculature origin. DAB immunohistochemistry of adult human hippocampus sections and immunocytochemistry of the isolated cells showing positive staining for prolyl-4-hyroxylase (P4H; A–D), Vimentin (E–H) and alpha-smooth muscle actin (Alpha-SMA; I-L). Higher magnification images show P4H to be localized in the pyramidal cells of the CA1 region (A, B; red arrow) and in the blood vessels throughout the section (A, C; white arrow). Vimentin was largely localized to the astrocytes in the white matter (including stratum radiatum; E-F; red arrow), and again, the blood vessels (E-G; white arrow). Alpha-SMA staining was only visible in the blood vessels (I-K; all arrows). Scale: (B-C, F-G, J-K)  = 60 µm, (D, H, L)  = 100 µm. (**M**) Comparative analysis of gene expression using q-RT-PCR for FbCs and fibroblast cells isolated and grown from the adult human brain and lung respectively. The level of expression was measured using a standard curve and normalized to a house-keeping gene, GAPDH. From the graph, it is evident that our brain-derived FbCs have a fibroblast-like gene expression profile that appears to be more vasculature cell-like than mescenchymal stem cell (MSC)-like.

To fully characterize the primary AhNPC cultures, the origin(s) of these brain-derived FbCs must be elucidated. To achieve this, various cell lineage markers (Table S1) were tested on the FbC cultures and compared to a fibroblast cell line derived from human lung (hLFb). The three most likely cell linages were tested; 1) mesenchymal stem cells (MSCs), 2) pericytes/blood vasculature cells, and 3) fibroblast cells. ICC and western blot analysis showed a low level of the mescenhymal/fibroblast cell marker Thy-1 in the FbCs compared to the hLFbs (Figure S3 J-L), while a fibroblast-specific antigen, S100A4, was much higher in the FbCs (Figure S3 G-I). A more detailed analysis of gene expression at the transcript level using q-RT-PCR (primers listed in Table S2) indicated that FbCs strongly expressed genes that were associated with fibroblast cells originating from brain vasculature, such as, PDGFR-β and α-smooth muscle actin (α-SMA; Figure 5 M). In agreement with the ICC and western results, transcript levels for MSC-related genes were relatively lower in FbCs compared to hLFbs, while S100A4 was at least 10 fold higher (Figure 5 M). Furthermore, immunohistochemical studies of adult human hippocampus sections used for our *in vitro* cultures also showed many of our FbC-markers (P4H, Vimentin, and α-SMA) staining the blood vessels, hence corroborating the likelihood that our FbCs are of blood vessel origin (Figure 5 A–L). Table 2 summarizes these results.

**Table 2:** Summary of the characteristics of brain-derived FbCs compared to the human lung-derived fibroblast cells.

Cell Lineage	Markers	Human Brain FbCs	Human Lung Fibroblasts
**MSCs**	Thy1/CD90	**−**	**+++**
	5′-Nucleotidase/CD73	**+**	**+++**
**Pericytes/Blood vessels**	PDGFRβ/CD140β	**++**	**++**
	α-SMA	**+++**	**++**
	Nestin	**++**	**++**
**Fibroblasts**	Prolyl-4-hydroxylase α/β	**+++**	**+++**
	S100A4	**+++**	**+**
	Vimentin	**++**	**++**
	Fibronectin	**+++**	**+++**

Arbitrary scale based on a combined observation of ICC, western blot and q-RT-PCR data.

**−**  =  not detected, **+**  =  small, **++**  =  medium, **+++**  =  high expression.

## Discussion

Recent developments have provided evidence for the existence of functional NPCs in the so-called neurogenic regions of the adult human brain [4], [5], [9], [11], [12], [23], [24], as previously described in rodents [3], [4], [5], [6], [13], [25], [26]. By combining live cell imaging, immunocytochemistry and electrophysiological techniques, this study provided further evidence for the existence of adult NPCs in the human brain. More importantly, this study provided for the first time, evidence for the progressive changes in the cellular composition that occur during prolonged primary human NPC cultures. This area has been largely neglected, despite the fact that knowledge of the cellular composition of a heterogeneous cellular population is vital for the interpretation and reproducibility of experiments. Moreover, special emphasis was given to characterising the mitotic populations, as they are the main source of confusion regarding cell identity in primary NPC cultures [21], [27].

### Primary Adult Human Brain Cell Cultures Give Rise to a Heterogeneous Population In Vitro

Primary cultures isolated from the adult human brain gave rise to a heterogeneous population of brain-derived post-mitotic and mitotic cells. Initially, the post-mitotic population comprised mostly mature astrocytes and microglia, but neurons were also present in varying amounts. However, they became rapidly diluted out with subsequent passages due to their post-mitotic nature. This is in agreement with previous reports where post-mitotic cells could only be cultured for a short period of time [21], [28]. The mitotic cells, when cultured from the neurogenic regions (SVZ and hippocampus), comprised two distinct populations.

### Two Distinct Mitotic Populations Exist in AhNPC Cultures

#### Adult human neural progenitor cells

Cultures from the neurogenic regions gave rise to mitotic cells that could be classified into two types based on their morphology and immunostaining properties. The first population only arose from cultures containing the ventricular regions and the hippocampus. Morphologically, they were bipolar in shape and had phase-bright somas. These cells frequently formed spheres, incorporated BrdU and were immunoreactive for the proliferative marker ki67. The same cells co-expressed Nestin and GFAP, which are markers widely used as indicators for AhNPCs, as they are thought to arise from proliferative astroglial-like cells (type B and C cells of the SVZ and type I cells of the SGZ) [24], [29], [30]. Some co-expressed PSA-NCAM and βIII-tubulin, which might indicate a population that has already undergone asymmetric division to give rise to proliferative neuroblasts. *In vivo,* they represent the migrating cells predestined to give rise to granule cell neurons and interneurons in the hippocampus and the olfactory bulbs, respectively [31], [32], [33]. As reported previously [11], spontaneous NSs arose during the earlier passages (P1– P4), and on many occasions, adhered back onto the monolayer of cells and started extending multiple neurite projections. As NS formation is a defining characteristic of neural stem cells *in vitro* [3], [9], [34], we also tested for NS formation by sub-plating cells onto non-adhesive surfaces at every passage from monolayer cultures. Spheres frequently arose from all passages; however, in concordance with our monolayer cultures, only the spheres from earlier passages (<P5) were capable of differentiating into cells of the CNS, and hence, only these could be classified as NSs. The NSs from earlier passages were capable of giving rise to tertiary spheres but not more. These results complement other studies that found adult NPCs *in vitro* had limited proliferative and differentiating ability [10], [12], [13], [19]. However, despite the loss of their multipotentiality and hence their NS status, their proliferative ability remained until at least passage 9 (12 months in culture; longest current culture time). These later passage cultures are likely to be predominantly comprised of FbCs and this will be discussed in further detail. Based on these observations, we highly recommend that NPC-based experiments be conducted between the 3–5 passage period.

Interestingly, a couple of groups have published results in which AhNPCs were cultured for greater than 20 months (>12 passages) [9], [11]. These reports each used contrasting culture protocols and our protocol was more comparable with Ayuso-Sacido et al. where conventional serum-free medium supplemented with growth factors was used [9]. Leonard et al. utilized a high serum containing, growth factor-free medium, which is widely used to culture primary microglia and astrocytes [21], [35] and is commonly used by many to differentiate the NPCs [12], [25], [26], [36]. Our attempts to reproduce the high-serum culture conditions were unsuccessful as the majority of our cells developed into FbCs with very limited, if any, spontaneous sphere formation. The reasons for these differences are unclear.

#### Brain-derived fibroblast-like cells (FbCs)

The second mitotic cell population that co-exists with the AhNPCs, but is not only limited to cultures of the neurogenic regions is the FbC population. We have previously characterized FbCs cultured in serum containing culture conditions that were primarily designed for maintaining primary astrocytes and microglia [21]. Here, the FbCs were highly proliferative, were morphologically large (>50 µm) and flat, appeared phase-dark under phase-contrast microscopy, were highly immunoreactive for the collagen synthesizing enzyme P4H and the extracellular matrix protein fibronectin, and were negative for endothelial markers CD34 and Factor VIII [21]. These fibroblast cell-like characteristics led us to classify these cells as FbCs [21]. In our AhNPC cultures, cells with indistinguishable immunoreactive characteristics were identified and despite their morphological differences (more bipolar and phase-bright), they were classified as FbCs. In addition, the present study also found FbCs to express markers associated with NPCs, such as Nestin and Sox-2. Based on these observations, we tested: 1) whether the morphological differences were due to our serum-free NPC culture conditions, and 2) whether the FbCs were actually another form of NPCs *in vitro*. To test this, FbCs cultured in serum-containing conditions were cultured in AhNPC culture conditions for a period of 2 weeks. By removing serum and supplementing the media with B27, EGF and FGF-2, their morphology changed from a flat phase-dark to more rounded, phase-bright bipolar cells, closely resembling those FbCs seen in AhNPC cultures. As these bipolar FbCs still retained their P4H and fibronectin immunoreactivity, we concluded that the morphological differences were due to the different culture conditions. Interestingly, accompanying the morphological changes was a significant increase in the expression of the NPC marker Nestin and the early neuronal marker, βIII-tubulin. The small but significant increase in Nestin suggests that NPC conditions may be driving these FbCs further towards a precursor-like de-differentiated state. The concurrent up-regulation of βIII-tubulin came as a surprise as it represented a more neuronally committed cell line. One explanation is that the increase is due to the presence of the growth factors EGF and FGF-2, as they lead to increases in pro-neural gene expression in non-neuronal cell types [37]. Indeed, βIII-tubulin expression was attenuated when the growth factors were removed from the media (data not shown). However, the same growth factors did not stimulate βIII-tubulin expression in the majority of AhNPCs (rather decreased it), suggesting that this is either a FbC-specific effect or that FbCs might also be trans-differentiating into cells of an early neuronal phenotype. In addition, when FbCs were cultured in non-adherent NS culture conditions, they also gave rise to clonal spheres that could be passaged into secondary spheres. This was surprising, as sphere-forming ability in our culture conditions has so far been largely associated with NSC/NPC, or cancer stem cells in others’ work [3], [9], [14], [38]. This is not the first time fibroblast cells have shown NPC-like characteristics *in vitro* [37], [39]; however, it is the first time these results have been described from cells derived from tissue of the adult human brain. Due to their brain tissue origin and the expression of NPC markers, FbCs have become an ambiguous cell type in NPC cultures, as illustrated by the fact that similar cells have been classified as both protoplasmic astrocytes [27] and NPCs [15]. Therefore, in order to further investigate the notion that FbCs could indeed have NPC-like properties, we compared their neurogenic capacity to AhNPCs under differentiating conditions.

### AhNPCs Differentiate into Immunologically and Physiologically Identifiable Brain Cells

When AhNPC cultures of earlier passages (<5 passages) were induced to differentiate, they gave rise to cells with distinct neuronal and astroglial characteristics. These cells were immunoreactive for neuronal βIII-tubulin, MAP2ab, and astroglial GFAP, but devoid of the proliferative marker ki67 and the NSC marker Nestin. We could not observe cells immunoreactive for oligodendrocyte marker RIP4. This was of no great surprise as others have also found oligodendrocyte differentiation to be the most challenging [9]. As reported by numerous NPC studies [11], [40] we also observed an intermediate cell type that was immunoreactive for two lineage-specific markers, βIII-tubulin and GFAP. Previous investigators have classified these cells as ‘asterons’ and described them as a trans-differentiated state of a neuron or an astrocyte [11], [40]. However, as we found a large percentage of asterons spreading out from the differentiating NSs and because neurons arise from GFAP positive cells *in vivo* [41], we suggest that asterons could be a population of semi-committed immature neurons.

Differentiated NSs were studied using whole-cell patch-clamp techniques to test for functional differentiation. In agreement with previous reports [12], [16], [42] AhNPC-derived neurons gradually developed neurophysiological properties, with cells recorded at earlier time points (<7 days) exhibiting immature passive membrane properties such as depolarized RMP and a relatively high R_m._ They also showed very limited active membrane properties with the occasional cell showing a very immature ‘depolarizing hump’ in response to strong depolarizing stimuli. These ‘depolarizing humps’ are likely to be a product of outward K^+^ current activated by membrane depolarization, as blocking voltage-gated K^+^ channels abolished this signal [42]. This K^+^ current is also observed in migrating neuroblasts of the RMS in adult rats [43], suggesting that it is channel activity characteristic of a developing and/or migrating neuron. By 3–4 weeks of differentiation, cells developed mature passive membrane properties with the average RMP and R_m_ at neurophysiological levels of −65±2.1 mV and 195±50 MΩ. Furthermore, 30% fired single immature action potentials (iAP) that were accompanied by fast-inactivating inward and slow-inactivating outward currents. These iAPs are likely to be caused by a combination of voltage-gated Ca^2+^ and not yet fully developed Na^+^ channels [42]. The large outward currents seen in voltage-clamp are likely due to the pipette solution containing potassium gluconate instead of CsCl typically used to measure inward sodium currents. However, unlike previous reports, mature APs with typical short-lasting, overshooting waveforms could not be observed in these cultures, suggesting perhaps that the culture period of 3–4 weeks was not sufficient for complete neuronal maturation in our conditions. The possibility that iAPs were of astrocyte origin [44] was ruled out on the basis that the astrocyte-like cells conveyed a significantly hyperpolarized RMP, lacked the ability to fire APs and labelled with GFAP, while the AP-firing cells labelled with βIII-tubulin. Therefore, this study convincingly demonstrated that the adult human brain SVZ and the dentate gyrus give rise to AhNPCs that could be propagated for up to 5 passages (∼ 6 months) without losing their bipotency to differentiate into functionally maturing neurons and astrocytes.

### FbCs Cultured in AhNPC Conditions do not Differentiate into Functional Brain Cells and therefore are not NPC by Nature

Despite the large phenotypic overlap between the AhNPCs and FbCs during their proliferative states, FbCs failed to give rise to morphologically, immunologically and electrophysiologically identifiable neurons or astrocytes. Although the differentiated FbCs showed small neurite extensions and expressed βIII-tubulin, they failed to acquire more mature neuronal or astrocytic markers such as MAP2 and GFAP. Furthermore, they showed no electrophysiological resemblance to neuronally differentiated AhNPCs. This led us to believe that AhNPCs and FbCs are two phenotypically similar but functionally distinct populations of proliferative cells *in* *vitro.*

### Possible Neurovasculature Origin of the FbCs

In depth characterization of the AhNPC culture system is vital in achieving a more reliable and reproducible experimental platform. Therefore, the origin(s) of FbCs were investigated based on the three most probable sources. First, mesenchymal stem/precursor cells from the blood [45], [46], [47], second, the perivascular/vascular cells in the brain parenchyma [48], [49], and third, the fibroblast cells from the leptomeninges or the surrounding support tissue [50], [51], [52], [53], [54]. The results showed that fibroblast-related genes in the FbCs, such as P4H, Vimentin and Nestin, were expressed at comparable levels to a control fibroblast cell line, reconfirming the highly fibroblast-like characteristics of these cells. In addition, our brain-derived FbCs highly expressed vasculature cell genes *PDGFRβ* and *α-SMA* [49], [55], but minimally expressed mesenchymal stem cell genes, *THY1/CD90* and *CD73* [56], [57]. Furthermore, many of FbC markers were localized to the blood vessels in the hippocampal sections used for our AhNPC cultures, although markers such as P4H and Vimentin also labelled neuronal and astrocytic cells respectively. Overall, these data indicate a neurovasculature origin of these FbCs. In fact, a cell type strongly resembling our FbCs has been described from the adult human spinal cord [48]. Although there are certain differences, such as their inability to form spheres under NPC culture conditions, when cultured under serum-containing conditions, these spinal cord cells behaved like our FbCs in that they proliferated as an adherent monolayer and expressed Nestin, Sox2, PDGFRβ and α-SMA. Furthermore, they also found these antigens to be localized to the blood vessels of the spinal cord [48]. Neurovasculature contamination of brain tissue cultures is unavoidable and when taken together with the above results, it appears our brain-derived FbCs are likely to be of neurovascular origin. Therefore, investigators are urged to correctly identify the FbCs, as they can cause considerable confusion in AhNPC cultures. However, when identified correctly, they may provide another neuroectodermally primed cell line for research.

### Limited Proliferative Ability of Biopsy-obtained AhNPCs In Vitro

In agreement with the majority of primary AhNPC culture studies [19], [20], [58], our results also demonstrated the limited proliferative ability and the relatively rapid loss of multipotentency seen with NPCs obtained from adult compared to those from embryonic or fetal brain tissue [59], [60]. This phenomenon can be explained by the fact that the number of ‘true’ neural stem cells are known to decrease during development and remain largely quiescent in adulthood until stimulated to proliferate [61]. These ‘slow dividing’ cells proliferate at a much slower rate and give rise to the rapidly amplifying NPCs, which contribute to the majority of the dividing cells *in vivo* [62] and the NS growth *in vitro* [34], [63]. In rodents, it has been reported that only the SVZ, and not the hippocampal dentate gyrus, is rich in ‘true’ NSCs [64]. As the majority of our cultures were from the hippocampal regions that had only a limited area of the SVZ lining the inferior horn of the lateral ventricle, it is likely that our tissue contained predominantly rapidly proliferating NPCs and very little, if any, ‘true’ NSCs. Another factor to consider is the potential impact of epileptic pathology on the process of neurogenesis, as the majority of our cultured brain tissues were obtained from temporal lobectomies for the treatment of mesial temporal lobe epilepsy. At least in animal models, epileptic development can largely be divided into two phases; acute, which accounts for the first couple of months after the initial seizure generating insult, and chronic, which accounts for the period succeeding the acute phase of the disease [65]. These distinct phases have very differing effects on neurogenesis. In general, the acute phase results in an increase in neurogenesis and neuronal differentiation [66], [67]. There is popular belief that this increase in neurogenesis leads to the generation of aberrant neuronal circuitry that is responsible for the generation of spontaneous recurrent seizures [66], [68]. However, during the chronic phase of the disease, studies generally report a decrease in neurogenesis, more specifically, in neuronal differentiation [69], [70]. Some studies associate this phenomenon with the deficits in hippocampal-associated learning tasks seen in animals and patients with chronic epilepsy [58], [69]. The majority of human studies rely on surgically obtained tissue, and since surgical intervention is usually the last mode of therapy, most cases can be regarded as chronic epilepsy. In agreement with animal studies, IHC analyses found no change or a decrease in adult hippocampal neurogenesis [71], [72]. As our cultures used the same tissue source as the study above, it is likely that our cultured hippocampal NPCs came from neurogenically-compromised tissue. This could account for the limited proliferative capacity and the relatively low rate of neuronal differentiation seen in not just our cultured NPCs, but also in other publications that use similar tissue sources [13], [19].

In summary, we have shown that neural progenitor cell cultures from the adult human brain generate a heterogeneous population of cells that gradually change in composition over several passages. We have demonstrated that there are two distinct mitotically active populations *in vitro,* the NPCs and the FbCs. Under NPC culture conditions, they showed remarkable phenotypic similarity; yet, they gave rise to very different cell types when differentiated. The NPCs gave rise to antigenically and functionally identifiable neurons and glia, while FbCs only expressed an immature neuronal marker. Therefore, it is clear that FbCs are not NPCs. However, FbCs, which might be from a neurovascular origin, do show a high degree of plasticity and a tendency towards a neuroectodermal lineage and these properties may benefit investigators by providing a readily accessible source for reprogrammable primary brain cells [73], [74], [75], [76]. As for the NPC cultures, unless new protocols are introduced that allow for the isolation and culture of ‘true’ NSCs from the adult human brain without genetic manipulation and increased cell viability, we recommend avoiding the earlier passages (<2, due to the residual primary astrocytes and neurons) and the later passages (>5, due to the FbCs) for conducting neurogenesis research. We believe these guidelines will allow investigators to conduct more reliable and reproducible AhNPC-based experiments and advance the field towards establishing a standardized adult human brain cell culture system.

## Materials and Methods

For detailed methods regarding many of the methodologies used in this study, please refer to [77].

### Tissue Dissociation and Culture

Biopsy human brain tissue containing the anterior temporal lobe and the hippocampus were obtained from surgery for medically refractory epilepsy. All specimens were collected with written patient consent and ethical approval from the Northern X Ethics Committee (biopsy tissue) and the University of Auckland Human Participants Ethics Committee. Eleven biopsy specimens (mean age 45 years) were collected from the surgical theatre at the Auckland City Hospital and transported to our laboratory in Ca^2+^ and Mg^2+^ free ice-cold Hank’s balanced salt solution (HBSS; Gibco). Tissue containing the periventricular zone and the hippocampus was dissected and dissociated prior to being digested in HBSS containing 2.5 U/mL papain (Worthington) and 100 U/mL DNase 1 (Invitrogen) for 20 minutes at 37°C with gentle rotation, which included a gentle trituration step at 10 minutes. Enzymatic digestion was halted by the addition of NPC proliferation media; DMEM:F12 containing B27 (Invitrogen), Penicillin/Streptomycin (Gibco), GlutaMAX (Invitrogen), 40 ng/mL FGF-2 (Peprotech), 40 ng/mL EGF (Peprotech) and 2 µg/mL Heparin (Sigma). Cells were collected by centrifugation (170 *g*×10 minutes), resuspended in the NPC proliferation media and plated onto un-coated T25 culture flasks (Nunc). The following day, culture flasks were gently agitated to detach any loosely adhered cells and all the media was collected and replated onto a fresh T25 culture flask. Media was half changed every 2–3 days and cultures were serially passaged every 20–30 days. Out of the 11 specimens, 9 showed sustained growth (>3 passages) in NPC proliferation media and were used for experimentation.

Human lung fibroblasts were cultured as previously published [21] as a monolayer on un-coated tissue culture flasks in DMEM:F12 base medium supplemented with 10% fetal bovine serum (Gibco), Penicillin/Streptomycin and GlutaMAX.

### Generation of Primary and Secondary Spheres

Adherent cultures frequently formed spontaneous spheres during expansion. To further promote sphere formation, cells were plated at 50,000 cells/mL in 100 mm bacteriological grade culture dishes (Falcon) in NPC proliferation media. Primary spheres generally formed within 1 week and continued to grow in size and number. Secondary and tertiary spheres were generated by dissociating the primary spheres into smaller spheres by trituration and replating them back into new culture dishes.

### Differentiation of NPCs into Neurons and Astrocytes

Whole spheres or single cells dissociated from the spheres were plated onto PDL/Laminin (Sigma) and the culture medium was changed from the NPC proliferation to the differentiation medium (DMEM:F12 containing 1% FBS, 40 ng/mL NGF and BDNF (Preprotech)).

### Immunocytochemistry

Cells were fixed in 4% paraformaldehyde (PFA) for 20 minutes at room temperature and permeabilized by 3×10 minute washes in PBS containing 0.1% Triton-X (PBS-T). Antibodies (Table S1) were dissolved in immunobuffer comprising PBS-T with merthiolate and 1% normal goat serum. Cells were stained with primary antibodies listed in Table S1 and incubated overnight at 4°C, then visualized after a 3-hour room temperature incubation with species-specific fluorescent (Alexa 488 or 594; Invitrogen) or biotin-conjugated (Sigma) secondary antibody. DAB immunoprecipitation was used to visualize biotin-conjugated secondary antibodies and all nuclei were counterstained with 20 µM Hoechst 33258 (Sigma).

### Immunohistochemistry

Protocols from Waldvogel et al. were used for handling and processing of donated brain tissue [78]. Briefly, the paraformaldehyde fixed tissue blocks were coronally sectioned into 50 µm sections and processed for immunohistochemistry as free-floating sections. In cases where antigen-retrieval was necessary, the sections were microwave-boiled in citrate buffer (pH 6.0) prior to being incubated for 3 days at 4°C with the antibodies listed in Table S1. Antibody binding was visualized using techniques described for immunocytochemistry section above and in Waldvogel et al. [78].

### BrdU Assay

10 µM BrdU (Roche) was added to the cultures 24 hours prior to the completion of an experiment. Cells were fixed in ice-cold methanol for 15 minutes at 4°C followed by a 45 minute incubation in 2 M HCl at 37°C. Wells were subsequently neutralized by washes in 0.1 M borate buffer pH 8.5 and PBS. Primary BrdU (1∶500; Roche) antibody was dissolved in PBS with 1% bovine serum albumin and incubated with cells overnight at 4°C. Primary antibody visualization was identical to that used for immunocytochemistry.

### Western Blot

Cell culture medium was removed and the cells were washed twice in ice-cold PBS. Protein lysates were prepared and western blots were performed by protocols previously described [79].

### Quantitative RT-PCR

Target gene expression levels were evaluated by quantitative RT-PCR using a 7900HT Fast Real Time PCR system (Applied Biosystems, Singapore). Total RNA was isolated at designated time points using RNeasy kit (Qiagen Inc.) and stored at −80°C until further use. cDNA synthesis was performed with SuperScript III first strand synthesis kit (Invitrogen) using approximately 3 µg of DNase I-treated (Promega) RNA, and subjected to q-RT-PCR using Platinum SYBR Green qPCR SuperMix-UDG with Rox kit (Invitrogen). The primers are detailed in Table S2 and the relative changes were analysed according to the ΔCT method [80]. Each PCR run included a negative RT and non-template control, as well as melting curve assays to confirm specific product amplification. The plotted data represent the mean values of at least 3 independent experiments ± standard error.

### Electrophysiology

Subsets of NSs were plated onto PDL/Laminin coated coverslips (Menzel-Glaser) and whole-cell patch-clamp technique was used to examine neurophysiological properties of individual cells as previously described [81], [82], [83]. Coverslips were placed in a recording chamber of an upright microscope (Olympus BX51) and perfused with artificial cerebral spinal fluid (150 mM NaCl, 4 mM KCl, 2 mM CaCl_2_, 2 mM MgCl_2_, 10 mM HEPES and 10 mM Glucose) at room temperature. Patch pipettes were fabricated from thick-walled borosilicate glass with an average resistance of 4–6 MΩ. Internal solution consisted of 120 mM K-Gluconate, 40 mM HEPES, 5 mM MgCl_2_, 2 mM NaATP, 0.3 mM NaGTP, pH 7.2 with KOH and Alexa 594 dye (Invitrogen) for post-patch identification of the cells. During the patch-clamping procedure, cells were visualised by infrared DIC microscopy. Current and voltage-clamp recordings were made using a Multiclamp 700A amplifier (Axon Instruments). Offline analysis was performed with Axograph 8 software (Axograph Scientific). In voltage clamp, fibre-bearing cells were held at a holding potential of −60 mV. Membrane resistance (R_m_), access resistance and cell capacitance were measured in voltage-clamp mode in response to a square depolarizing pulse of 20 mV at 100 Hz. In current-clamp mode, resting membrane potentials (RMPs) were recorded at zero holding current and membrane responses were examined by multiple current steps of 30 pA at 1 Hz moving the cell from −30 pA to 150 pA. Under voltage-clamp, active membrane properties were tested by applying 10 mV steps at 1 Hz, taking the membrane potential of the cell from −70 mV to 20 mV.

The results are presented as a mean ± SEM for all the recorded membrane properties and passive membrane properties were statistically tested by using independent sample *t-*tests for a significance value of P<0.05. The recorded coverslips were fixed as described in the ‘immunocytochemistry’ section above and the antigenicity of the recorded cells was visualized using fluorescent microscopy (Leica DMIRB, Germany).

## Supporting Information

Figure S1 **Post-mitotic cells isolated from the SVZ region of adult human brain specimens.** Isolated cells were grown in serum-free NPC proliferation media and fixed after 2 weeks *in vitro.* (**A-C**) Arrows show ki67 negative GFAP positive cells, indicative of a post-mitotic astrocyte. (**D-F**) Arrows show a βIII-tubulin positive, GFAP negative neuron. (**G-I**) Arrows show CD45 and PU1 positive microglia. Scale: 100 µm.
(TIF)

Figure S2 **Mitotically active cells isolated from the SVZ region of adult human brain specimens.** Isolated cells were grown in serum-free NPC proliferation media and fixed after 2 weeks *in vitro*. (**A-C**) White arrows show Nestin and GFAP positive cells, while the red arrows show Nestin only cells. (**D-F**) Arrows show proliferative ki67 positive, GFAP positive cells, and (**G-I**) show BrdU incorporated PSA-NCAM and βIII-tubulin cells. (**A-I**) Collectively show the presence of NPCs. (**J-L**) Arrows show Nestin and ki67 positive cells and in (**M-P**), arrows show the same population of cells that are prolyl-4-hydroxylase (P4H) and fibronectin (FN) positive. (**J-P**) Collectively show brain-derived FbCs. Scale: 100 µm.
(TIF)

Figure S3 **Antigenicity profile of brain-derived FbCs compared to fibroblast cells isolated from the human lung.** Immunocytochemistry (ICC) and western blot showing strong expression of P4H **(A-C)** and Vimentin **(D-F)** in both cell types. However, S100A4 (fibroblast-specific antigen) showed higher protein levels in brain-derived FbCs compared to those isolated from the lung **(G-I).** Thy-1/CD90 was the opposite, being undetectable in the brain-derived FbCs while highly expressed in the lung-derived cells **(J-L).** Alpha-SMA and Nestin both showed similar protein levels in both cultures. Scale: 100 µm. Please note that Thy-1 antibody labelled a very definite band at the correct molecular weight on a western blot and the level of expression strongly correlated with gene expression data (q-RT-PCR). However, ICC results continually showed non-specific nuclear staining (J-K), and hence it was concluded that our antibody did not work on our current 4% PFA-fixed cells.
(TIF)

Table S1 **Antibodies used and their dilutions.**
(DOC)

Table S2 **List and sequence of primers used for RT-PCR experiments.**
(DOC)

## References

[1] Altman J (1962). Are new neurons formed in the brains of adult mammals?. Science.

[2] Altman J, Das GD (1965). Post-natal origin of microneurones in the rat brain.. Nature.

[3] Reynolds BA, Weiss S (1992). Generation of neurons and astrocytes from isolated cells of the adult mammalian central nervous system.[see comment].. Science.

[4] Eriksson PS, Perfilieva E, Bjork-Eriksson T, Alborn AM, Nordborg C (1998). Neurogenesis in the adult human hippocampus.[see comment].. Nature Medicine.

[5] Curtis MA, Kam M, Nannmark U, Anderson MF, Axell MZ (2007). Human neuroblasts migrate to the olfactory bulb via a lateral ventricular extension.. Science.

[6] Doetsch F, Alvarez-Buylla A (1996). Network of tangential pathways for neuronal migration in adult mammalian brain.. Proceedings of the National Academy of Sciences of the United States of America.

[7] Lois C, Garcia-Verdugo JM, Alvarez-Buylla A (1996). Chain migration of neuronal precursors.. Science.

[8] Kukekov VG, Laywell ED, Suslov O, Davies K, Scheffler B (1999). Multipotent stem/progenitor cells with similar properties arise from two neurogenic regions of adult human brain.. Exp Neurol.

[9] Ayuso-Sacido A, Roy NS, Schwartz TH, Greenfield JP, Boockvar JA (2008). Long-term expansion of adult human brain subventricular zone precursors.. Neurosurgery 62: 223–229; discussion.

[10] Johansson CB, Svensson M, Wallstedt L, Janson AM, Frisen J (1999). Neural stem cells in the adult human brain.. Exp Cell Res.

[11] Leonard BW, Mastroeni D, Grover A, Liu Q, Yang K (2009). Subventricular zone neural progenitors from rapid brain autopsies of elderly subjects with and without neurodegenerative disease.. J Comp Neurol.

[12] Moe MC, Westerlund U, Varghese M, Berg-Johnsen J, Svensson M (2005). Development of neuronal networks from single stem cells harvested from the adult human brain.. Neurosurgery 56: 1182–1188; discussion.

[13] Palmer TD, Schwartz PH, Taupin P, Kaspar B, Stein SA (2001). Cell culture. Progenitor cells from human brain after death.. Nature.

[14] Richardson RM, Holloway KL, Bullock MR, Broaddus WC, Fillmore HL (2006). Isolation of neuronal progenitor cells from the adult human neocortex.. Acta Neurochir (Wien).

[15] Walton NM, Sutter BM, Chen HX, Chang LJ, Roper SN (2006). Derivation and large-scale expansion of multipotent astroglial neural progenitors from adult human brain.. Development.

[16] Westerlund U, Moe MC, Varghese M, Berg-Johnsen J, Ohlsson M (2003). Stem cells from the adult human brain develop into functional neurons in culture.. Experimental Cell Research.

[17] Rietze RL, Reynolds BA (2006). Neural Stem Cell Isolation and Characterization..

[18] Sanai N, Tramontin AD, Quinones-Hinojosa A, Barbaro NM, Gupta N (2004). Unique astrocyte ribbon in adult human brain contains neural stem cells but lacks chain migration.[see comment].. Nature.

[19] Nunes MC, Roy NS, Keyoung HM, Goodman RR, McKhann G, 2nd (2003). Identification and isolation of multipotential neural progenitor cells from the subcortical white matter of the adult human brain.. Nature Medicine.

[20] Arsenijevic Y, Villemure JG, Brunet JF, Bloch JJ, Deglon N (2001). Isolation of multipotent neural precursors residing in the cortex of the adult human brain.. Exp Neurol.

[21] Gibbons HM, Hughes SM, Van Roon-Mom W, Greenwood JM, Narayan PJ (2007). Cellular composition of human glial cultures from adult biopsy brain tissue.. J Neurosci Methods.

[22] Bifari F, Decimo I, Chiamulera C, Bersan E, Malpeli G (2009). Novel stem/progenitor cells with neuronal differentiation potential reside in the leptomeningeal niche.. J Cell Mol Med.

[23] Jin K, Wang X, Xie L, Mao XO, Zhu W (2006). Evidence for stroke-induced neurogenesis in the human brain.. PNAS.

[24] Curtis MA, Penney EB, Pearson AG, van Roon-Mom WMC, Butterworth NJ (2003). Increased cell proliferation and neurogenesis in the adult human Huntington’s disease brain.. Proceedings of the National Academy of Sciences of the United States of America.

[25] Roy NS, Benraiss A, Wang S, Fraser RA, Goodman R (2000). Promoter-targeted selection and isolation of neural progenitor cells from the adult human ventricular zone.. J Neurosci Res.

[26] Roy NS, Wang S, Jiang L, Kang J, Benraiss A (2000). In vitro neurogenesis by progenitor cells isolated from the adult human hippocampus.[see comment].. Nature Medicine.

[27] Lue LF, Brachova L, Walker DG, Rogers J (1996). Characterization of glial cultures from rapid autopsies of Alzheimer’s and control patients.. Neurobiol Aging.

[28] Brewer GJ, Espinosa J, McIlhaney MP, Pencek TP, Kesslak JP (2001). Culture and regeneration of human neurons after brain surgery.. J Neurosci Methods.

[29] Doetsch F, Caille I, Lim DA, Garcia-Verdugo JM, Alvarez-Buylla A (1999). Subventricular zone astrocytes are neural stem cells in the adult mammalian brain.. Cell.

[30] Quinones-Hinojosa A, Sanai N, Soriano-Navarro M, Gonzalez-Perez O, Mirzadeh Z (2006). Cellular composition and cytoarchitecture of the adult human subventricular zone: a niche of neural stem cells.. J Comp Neurol.

[31] Hu H, Tomasiewicz H, Magnuson T, Rutishauser U (1996). The role of polysialic acid in migration of olfactory bulb interneuron precursors in the subventricular zone.. Neuron.

[32] Kuhn HG, Dickinson-Anson H, Gage FH (1996). Neurogenesis in the dentate gyrus of the adult rat: age-related decrease of neuronal progenitor proliferation.. Journal of Neuroscience.

[33] van Praag H, Schinder AF, Christie BR, Toni N, Palmer TD (2002). Functional neurogenesis in the adult hippocampus.. Nature.

[34] Louis SA, Rietze RL, Deleyrolle L, Wagey RE, Thomas TE (2008). Enumeration of neural stem and progenitor cells in the neural colony-forming cell assay.. Stem Cells.

[35] De Groot CJ, Langeveld CH, Jongenelen CA, Montagne L, Van Der Valk P (1997). Establishment of human adult astrocyte cultures derived from postmortem multiple sclerosis and control brain and spinal cord regions: immunophenotypical and functional characterization.. J Neurosci Res.

[36] Pagano SF, Impagnatiello F, Girelli M, Cova L, Grioni E (2000). Isolation and characterization of neural stem cells from the adult human olfactory bulb.. Stem Cells.

[37] Joannides A, Gaughwin P, Scott M, Watt S, Compston A (2003). Postnatal astrocytes promote neural induction from adult human bone marrow-derived stem cells.. J Hematother Stem Cell Res.

[38] Laks DR, Masterman-Smith M, Visnyei K, Angenieux B, Orozco NM (2009). Neurosphere formation is an independent predictor of clinical outcome in malignant glioma.. Stem Cells.

[39] Rieske P, Krynska B, Azizi SA (2005). Human fibroblast-derived cell lines have characteristics of embryonic stem cells and cells of neuro-ectodermal origin.. Differentiation.

[40] Laywell ED, Kearns SM, Zheng T, Chen KA, Deng J (2005). Neuron-to-astrocyte transition: phenotypic fluidity and the formation of hybrid asterons in differentiating neurospheres.. J Comp Neurol.

[41] Kriegstein A, Alvarez-Buylla A (2009). The glial nature of embryonic and adult neural stem cells.. Annu Rev Neurosci.

[42] Moe MC, Varghese M, Danilov AI, Westerlund U, Ramm-Pettersen J (2005). Multipotent progenitor cells from the adult human brain: neurophysiological differentiation to mature neurons.. Brain.

[43] Belluzzi O, Benedusi M, Ackman J, LoTurco JJ (2003). Electrophysiological differentiation of new neurons in the olfactory bulb.. J Neurosci.

[44] Sontheimer H, Black JA, Ransom BR, Waxman SG (1992). Ion channels in spinal cord astrocytes in vitro. I. Transient expression of high levels of Na+ and K+ channels.. J Neurophysiol.

[45] Bucala R, Spiegel LA, Chesney J, Hogan M, Cerami A (1994). Circulating fibrocytes define a new leukocyte subpopulation that mediates tissue repair.. Mol Med.

[46] Mori L, Bellini A, Stacey MA, Schmidt M, Mattoli S (2005). Fibrocytes contribute to the myofibroblast population in wounded skin and originate from the bone marrow.. Exp Cell Res.

[47] Hirohata S, Yanagida T, Nagai T, Sawada T, Nakamura H (2001). Induction of fibroblast-like cells from CD34(+) progenitor cells of the bone marrow in rheumatoid arthritis.. J Leukoc Biol.

[48] Mamaeva D, Ripoll C, Bony C, Teigell M, Perrin FE (2011). Isolation of mineralizing Nestin+ Nkx6.1+ vascular muscular cells from the adult human spinal cord.. BMC Neurosci.

[49] Bondjers C, He L, Takemoto M, Norlin J, Asker N (2006). Microarray analysis of blood microvessels from PDGF-B and PDGF-Rbeta mutant mice identifies novel markers for brain pericytes.. FASEB J.

[50] Haines DE, Harkey HL, al-Mefty O (1993). The “subdural” space: a new look at an outdated concept.. Neurosurgery.

[51] Blass JP, Markesbery WR, Ko LW, DeGiorgio L, Sheu KF (1994). Presence of neuronal proteins in serially cultured cells from autopsy human brain.. J Neurol Sci.

[52] DeGiorgio LA, Bernstein JJ, Blass JP (1997). Implantation of cultured human leptomeningeal cells into rat brain.. Int J Dev Neurosci.

[53] DeGiorgio LA, Sheu KF, Blass JP (1994). Culture from human leptomeninges of cells containing neurofilament protein and neuron-specific enolase.. J Neurol Sci.

[54] Black RS, DeGiorgio LA, Sheu KF, Darzynkiewicz Z, Duffy JT (1994). Expression of neuronal proteins in cells from normal adult rat brain propagated in serial culture.. J Neurochem.

[55] Arimura K, Ago T, Kamouchi M, Nakamura K, Ishitsuka K (2012). PDGF Receptor beta Signaling in Pericytes Following Ischemic Brain Injury.. Curr Neurovasc Res.

[56] Hombach-Klonisch S, Panigrahi S, Rashedi I, Seifert A, Alberti E (2008). Adult stem cells and their trans-differentiation potential–perspectives and therapeutic applications.. J Mol Med (Berl).

[57] Rege TA, Hagood JS (2006). Thy-1, a versatile modulator of signaling affecting cellular adhesion, proliferation, survival, and cytokine/growth factor responses.. Biochim Biophys Acta.

[58] Coras R, Siebzehnrubl FA, Pauli E, Huttner HB, Njunting M (2010). Low proliferation and differentiation capacities of adult hippocampal stem cells correlate with memory dysfunction in humans..

[59] Vescovi AL, Gritti A, Galli R, Parati EA (1999). Isolation and intracerebral grafting of nontransformed multipotential embryonic human CNS stem cells.. J Neurotrauma.

[60] Vescovi AL, Parati EA, Gritti A, Poulin P, Ferrario M (1999). Isolation and cloning of multipotential stem cells from the embryonic human CNS and establishment of transplantable human neural stem cell lines by epigenetic stimulation.. Exp Neurol.

[61] Kazanis I, Lathia JD, Vadakkan TJ, Raborn E, Wan R (2010). Quiescence and activation of stem and precursor cell populations in the subependymal zone of the mammalian brain are associated with distinct cellular and extracellular matrix signals.. J Neurosci.

[62] Doetsch F, Garcia-Verdugo JM, Alvarez-Buylla A (1999). Regeneration of a germinal layer in the adult mammalian brain.. Proceedings of the National Academy of Sciences of the United States of America.

[63] Reynolds BA, Rietze RL (2005). Neural stem cells and neurospheres–re-evaluating the relationship.. Nat Methods.

[64] Seaberg RM, van der Kooy D (2002). Adult rodent neurogenic regions: the ventricular subependyma contains neural stem cells, but the dentate gyrus contains restricted progenitors.. J Neurosci.

[65] Liu YW, Mee EW, Bergin P, Teoh HH, Connor B (2007). Adult neurogenesis in mesial temporal lobe epilepsy: a review of recent animal and human studies.. Curr Pharm Biotechnol.

[66] Parent JM, Elliott RC, Pleasure SJ, Barbaro NM, Lowenstein DH (2006). Aberrant seizure-induced neurogenesis in experimental temporal lobe epilepsy.. Ann Neurol.

[67] Bonde S, Ekdahl CT, Lindvall O (2006). Long-term neuronal replacement in adult rat hippocampus after status epilepticus despite chronic inflammation.. Eur J Neurosci.

[68] Jessberger S, Zhao C, Toni N, Clemenson GD, Li Y (2007). Seizure-associated, aberrant neurogenesis in adult rats characterized with retrovirus-mediated cell labeling.. J Neurosci.

[69] Hattiangady B, Rao MS, Shetty AK (2004). Chronic temporal lobe epilepsy is associated with severely declined dentate neurogenesis in the adult hippocampus.. Neurobiol Dis.

[70] Hattiangady B, Shetty AK (2010). Decreased neuronal differentiation of newly generated cells underlies reduced hippocampal neurogenesis in chronic temporal lobe epilepsy.. Hippocampus.

[71] D’Alessio L, Konopka H, Lopez EM, Seoane E, Consalvo D (2010). Doublecortin (DCX) immunoreactivity in hippocampus of chronic refractory temporal lobe epilepsy patients with hippocampal sclerosis.. Seizure.

[72] Liu YW, Curtis MA, Gibbons HM, Mee EW, Bergin PS (2008). Doublecortin expression in the normal and epileptic adult human brain.. Eur J Neurosci.

[73] Inoue H (2010). Neurodegenerative disease-specific induced pluripotent stem cell research..

[74] Takahashi K, Yamanaka S (2006). Induction of pluripotent stem cells from mouse embryonic and adult fibroblast cultures by defined factors.. Cell.

[75] Kim JB, Greber B, Arauzo-Bravo MJ, Meyer J, Park KI (2009). Direct reprogramming of human neural stem cells by OCT4.. Nature.

[76] Kim D, Kim CH, Moon JI, Chung YG, Chang MY (2009). Generation of human induced pluripotent stem cells by direct delivery of reprogramming proteins.. Cell Stem Cell.

[77] Park IHT, Waldvogel HJ, Montgomery JM, Mee EW, Bergin PS, Deleyrolle L, Reynolds BA Identifying neural progenitor cells in the adult human brain.. Methods Mol Biol: Humana Press.

[78] Waldvogel HJ, Curtis MA, Baer K, Rees MI, Faull RL (2006). Immunohistochemical staining of post-mortem adult human brain sections.. Nat Protoc.

[79] Gibbons H, Sato TA, Dragunow M (2003). Hypothermia suppresses inducible nitric oxide synthase and stimulates cyclooxygenase-2 in lipopolysaccharide stimulated BV-2 cells.. Brain Res Mol Brain Res.

[80] Livak KJ, Schmittgen TD (2001). Analysis of relative gene expression data using real-time quantitative PCR and the 2(-Delta Delta C(T)) Method.. Methods.

[81] Montgomery JM, Pavlidis P, Madison DV (2001). Pair recordings reveal all-silent synaptic connections and the postsynaptic expression of long-term potentiation.. Neuron.

[82] Emond MR, Montgomery JM, Huggins ML, Hanson JE, Mao L (2010). AMPA receptor subunits define properties of state-dependent synaptic plasticity.. J Physiol.

[83] Waites CL, Specht CG, Hartel K, Leal-Ortiz S, Genoux D (2009). Synaptic SAP97 isoforms regulate AMPA receptor dynamics and access to presynaptic glutamate.. J Neurosci.

