# Supplementary figures and images for: Adult Human Brain Neural Progenitor Cells (NPCs) and Fibroblast-Like Cells Have Similar Properties In Vitro but Only NPCs Differentiate into Neurons
Source: PLoS One. 2012 Jun 4;7(6):e37742. doi: 10.1371/journal.pone.0037742 (PMC3366988; doi:10.1371/journal.pone.0037742)

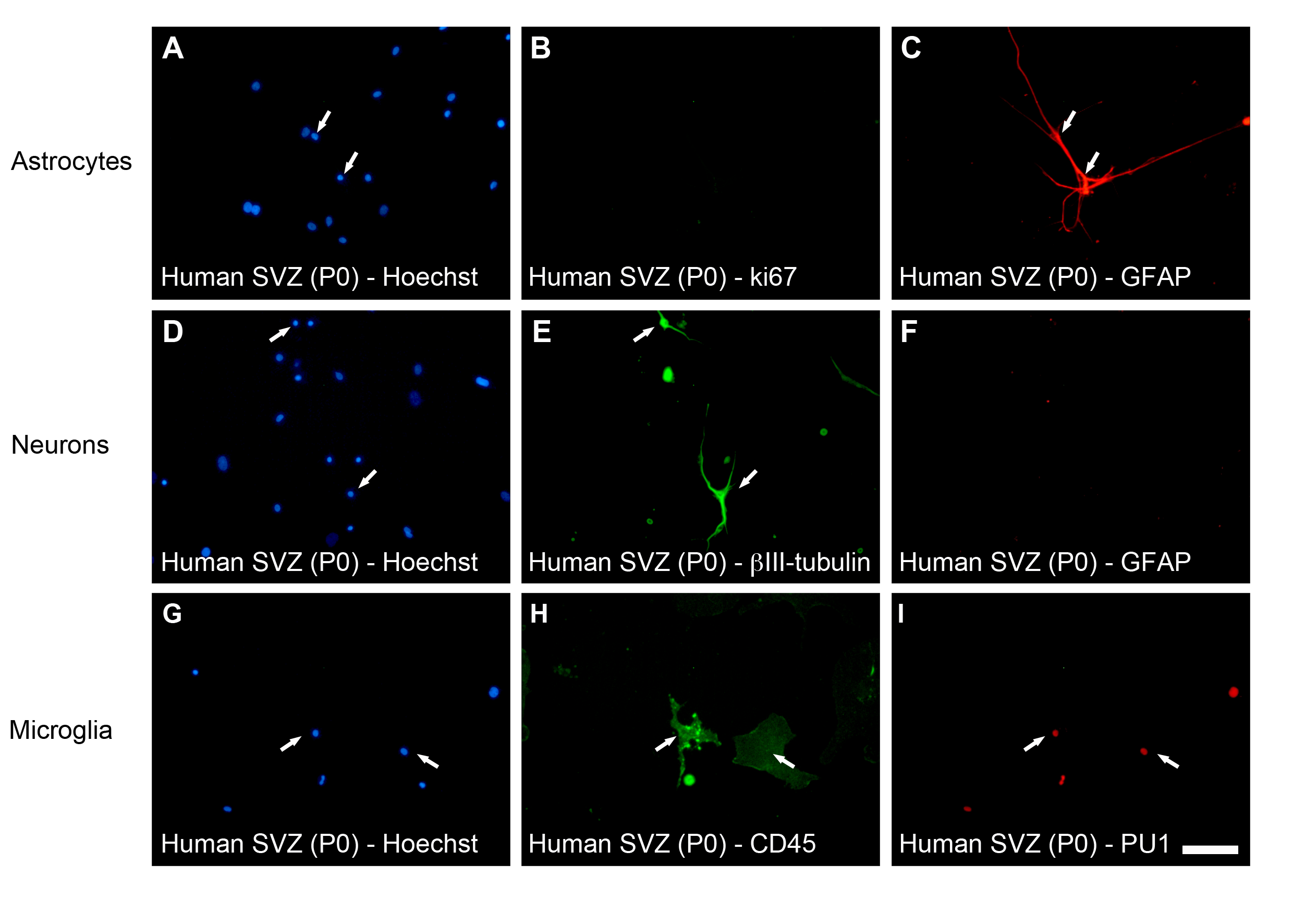

Supplement: Figure S1 — Post-mitotic cells isolated from the SVZ region of adult human brain specimens. Isolated cells were grown in serum-free NPC proliferation media and fixed after 2 weeks in vitro. (A-C) Arrows show ki67 negative GFAP positive cells, indicative of a post-mitotic astrocyte. (D-F) Arrows show a βIII-tubulin positive, GFAP negative neuron. (G-I) Arrows show CD45 and PU1 positive microglia. Scale: 100 µm. (TIF) [file pone.0037742.s001.tif]

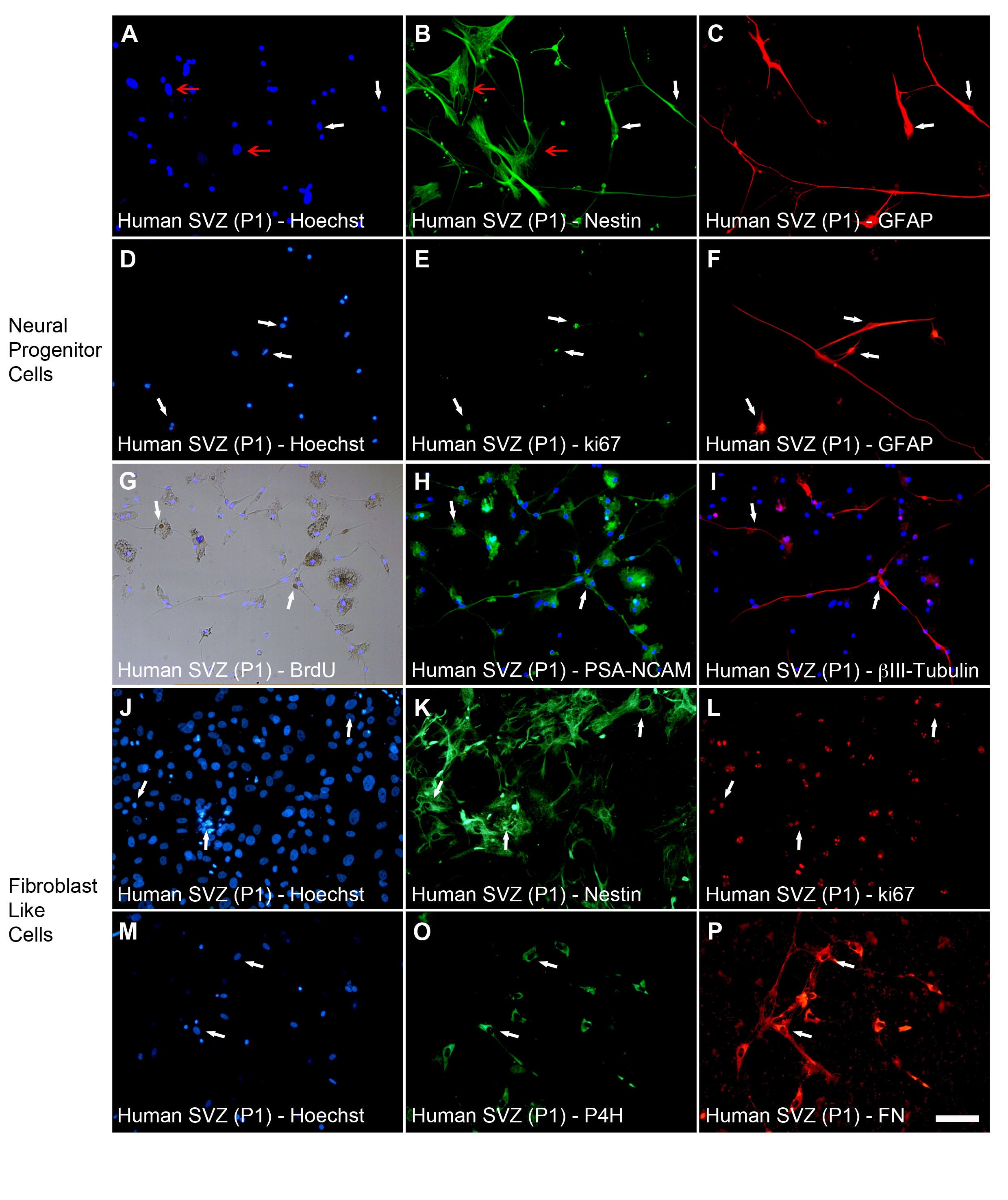

Supplement: Figure S2 — Mitotically active cells isolated from the SVZ region of adult human brain specimens. Isolated cells were grown in serum-free NPC proliferation media and fixed after 2 weeks in vitro. (A-C) White arrows show Nestin and GFAP positive cells, while the red arrows show Nestin only cells. (D-F) Arrows show proliferative ki67 positive, GFAP positive cells, and (G-I) show BrdU incorporated PSA-NCAM and βIII-tubulin cells. (A-I) Collectively show the presence of NPCs. (J-L) Arrows show Nestin and ki67 positive cells and in (M-P), arrows show the same population of cells that are prolyl-4-hydroxylase (P4H) and fibronectin (FN) positive. (J-P) Collectively show brain-derived FbCs. Scale: 100 µm. (TIF) [file pone.0037742.s002.tif]

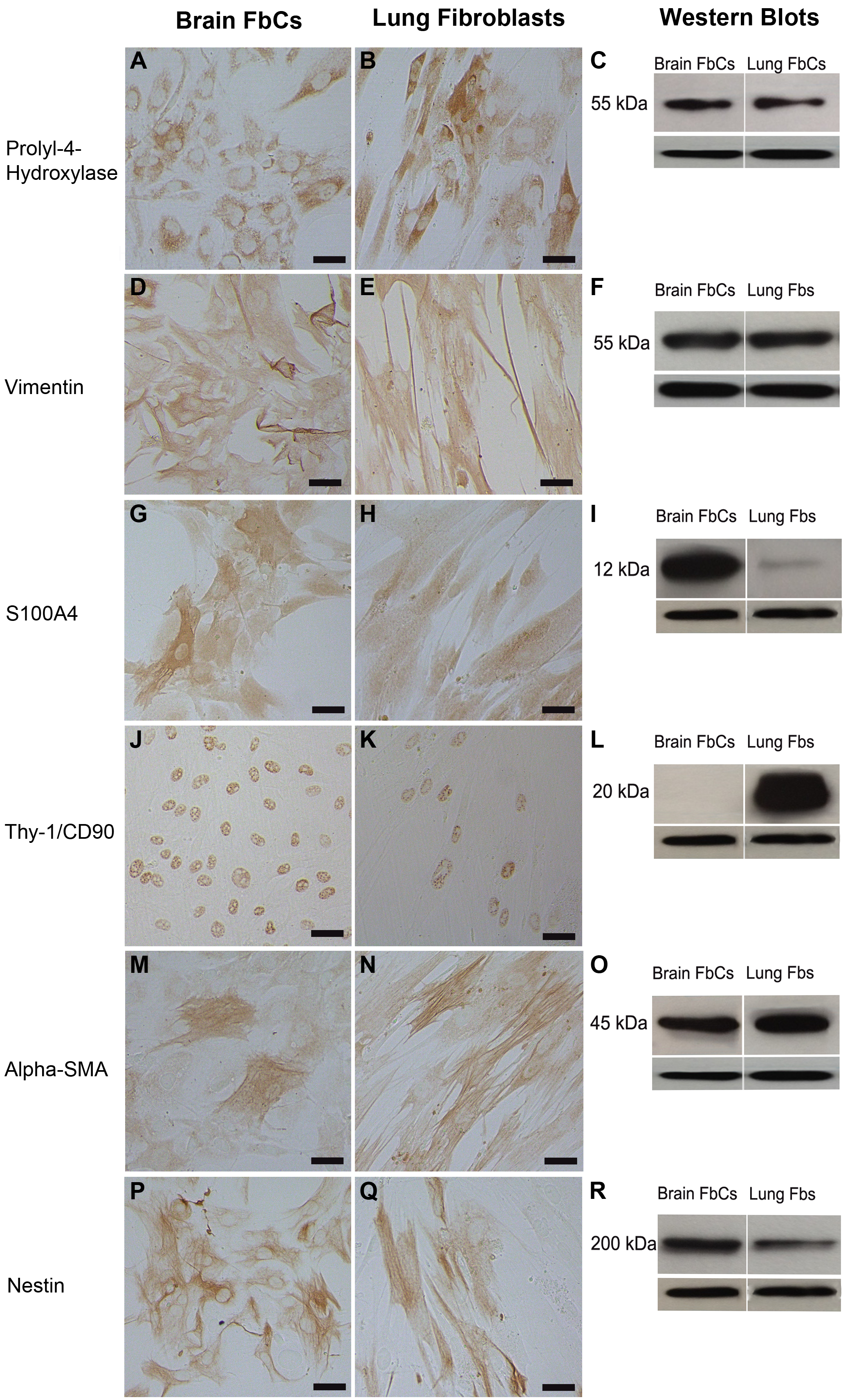

Supplement: Figure S3 — Antigenicity profile of brain-derived FbCs compared to fibroblast cells isolated from the human lung. Immunocytochemistry (ICC) and western blot showing strong expression of P4H (A-C) and Vimentin (D-F) in both cell types. However, S100A4 (fibroblast-specific antigen) showed higher protein levels in brain-derived FbCs compared to those isolated from the lung (G-I). Thy-1/CD90 was the opposite, being undetectable in the brain-derived FbCs while highly expressed in the lung-derived cells (J-L). Alpha-SMA and Nestin both showed similar protein levels in both cultures. Scale: 100 µm. Please note that Thy-1 antibody labelled a very definite band at the correct molecular weight on a western blot and the level of expression strongly correlated with gene expression data (q-RT-PCR). However, ICC results continually showed non-specific nuclear staining (J-K), and hence it was concluded that our antibody did not work on our current 4% PFA-fixed cells. (TIF) [file pone.0037742.s003.tif]
