# Supplementary material for: Adult Human Brain Neural Progenitor Cells (NPCs) and Fibroblast-Like Cells Have Similar Properties In Vitro but Only NPCs Differentiate into Neurons
Source: PLoS One. 2012 Jun 4;7(6):e37742. doi: 10.1371/journal.pone.0037742 (PMC3366988; doi:10.1371/journal.pone.0037742)
Supplement: Table S1 — Antibodies used and their dilutions. (DOC) [file pone.0037742.s004.doc]

**Table S1.** Antibodies used and their dilutions

| **Antibody** | **Host** | **Dilution** | **Source/Catalog Number** |
| --- | --- | --- | --- |
| **βIII-tubulin** | Mouse monoclonal | 1:1000 | Sigma/055k4771 |
| **BrdU** | Mouse monoclonal | 1:500 | Roche/BMC91318 |
| **CD45** | Mouse monoclonal | 1:500 | Abcam/ab8216 |
| **Fibronectin** | Rabbit polyclonal | 1:1000 | Dako/A0245 |
| **GFAP** | Mouse monoclonal | 1:2000 | Cell Signalling/#3670X |
| **GFAP** | Rabbit polyclonal | 1:2000 | Dako/Z0334 |
| **ki67** | Mouse monoclonal | 1:250 | Dako/M7240 |
| **ki67** | Rabbit polyclonal | 1:250 | Dako/A0047 |
| **MAP2ab** | Mouse monoclonal | 1:1000 | Sigma/M4403 |
| **Nestin** | Mouse monoclonal | 1:500 | Chemicon/MAB5326 |
| **Prolyl-4-hydroxylase β** | Mouse monoclonal | 1:1000 | Dako/M0877 |
| **PSA-NCAM** | Mouse monoclonal | 1:500 | Chemicon/MAB5324 |
| **PU1** | Rabbit polyclonal | 1:500 | Cell Signalling/#2258 |
| **Sox-2** | Mouse monoclonal | 1:500 | R&D/MAB 2018 |
| **Vimentin** | Rabbit polyclonal | 1:500 | Abcam/ab15248-1 |
| **α-SMA** | Mouse monoclonal | 1:1 | Dako/N1584 |
| **S100A4** | Mouse monoclonal | 1:200 | Abcam/ab27957 |
| **Thy1/CD90** | Rabbit monoclonal | 1:100 | Abcam/ab92574 |
| **PDGFRβ** | Rabbit polyclonal | 1:100 | Cell Signaling/#3168 |
