# Supplementary material for: Adult Human Brain Neural Progenitor Cells (NPCs) and Fibroblast-Like Cells Have Similar Properties In Vitro but Only NPCs Differentiate into Neurons
Source: PLoS One. 2012 Jun 4;7(6):e37742. doi: 10.1371/journal.pone.0037742 (PMC3366988; doi:10.1371/journal.pone.0037742)
Supplement: Table S2 — List and sequence of primers used for RT-PCR experiments. (DOC) [file pone.0037742.s005.doc]

**Table S2.** List and sequence of primers used for RT-PCR experiments

| **GENE** |  | **Sequence** | **Start bp** | **Stop bp** | **Amplicon length** |
| --- | --- | --- | --- | --- | --- |
| ***β-ACTIN* (h)** | Fw | AGGCCAACCGTGAAAAGATG | 338 | 357 | 79 bp |
|  | Rv | CACAGCCTGGATGGCTACGT | 417 | 398 |  |
| ***βIII-TUBULIN* (h)** | Fw | CAAGTTCTGGGAAGTCATCAGTGA | 54 | 77 | 68 bp |
|  | Rv | CCGAGTCGCCCACGTAGTT | 121 | 103 |  |
| ***NESTIN* (h)** | Fw | GCCGGCGTCTTGGATCT | 182 | 198 | 92 bp |
|  | Rv | AAAGGCTGGCACAGGTGTCT | 273 | 254 |  |
| ***GFAP* (h)** | Fw | GAGATCCGCACGCAGTATGA | 709 | 728 | 73 bp |
|  | Rv | ACTTGGAGCGGTACCACTCTTC | 781 | 760 |  |
| ***GAPDH* (h)** | Fw | CATGAGAAGTATGACAACAGCCT | 409 | 431 | 112 bp |
|  | Rv | AGTCCTTCCACGATACCAAAGT | 521 | 500 |  |
| ***THY1/CD90* (h)** | Fw | AGCAAGGACGAGGGCACCTACA | 286 | 307 | 68 bp |
|  | Rv | TGGGAGGAGATGGGTGGGGAAT | 353 | 332 |  |
| ***CD73* (h)** | Fw | ATGAACGCCCTGCGCTACGA | 313 | 332 | 78 bp |
|  | Rv | TGGCTCGATCAGTCCTTCCACACC | 390 | 367 |  |
| ***PDGFRβ* (h)** | Fw | CGCAAAGAAAGTGGGCGGCT | 751 | 770 | 80 bp |
|  | Rv | TGCAGGATGGAGCGGATGTGGT | 830 | 809 |  |
| ***α-SMA* (h)** | Fw | ACGTGGGTGACGAAGCACAGA | 164 | 184 | 84 bp |
|  | Rv | CGTCCCAGTTGGTGATGATGCC | 247 | 226 |  |
| ***S100A4* (h)** | Fw | GCCCAGCTTCTTGGGGAAAAGGA | 126 | 148 | 84 bp |
|  | Rv | ACCTCGTTGTCCCTGTTGCTGT | 209 | 188 |  |
